# *N*-[Bis(morpholin-4-yl)phosphino­yl]-2-chloro-2,2-difluoro­acetamide

**DOI:** 10.1107/S1600536811030194

**Published:** 2011-07-30

**Authors:** Mehrdad Pourayoubi, Anahid Saneei

**Affiliations:** a Department of Chemistry, Ferdowsi University of Mashhad, Mashhad 91779, Iran

## Abstract

The asymmetric unit of the title compound, C_10_H_17_ClF_2_N_3_O_4_P, consists of two independent mol­ecules in each of which the P atom adopts a distorted tetra­hedral environment with the P=O and N—H units in a *syn* orientation with respect to one another. Both morpholine rings in one of the phospho­ramide mol­ecules are disordered over two sets of sites, with site occupancies of 0.766 (7) and 0.234 (7) for one ring and 0.639 (10) and 0.361 (10) for the other. In the second phospho­ramide mol­ecule, one of the NC_4_H_8_O moieties is disordered over two sets of sites with site occupancies of 0.807 (6) and 0.193 (6). In the crystal, pairs of inter­molecular N—H⋯O(P) hydrogen bonds form two independent centrosymmetric dimers.

## Related literature

For patterns of hydrogen bonds in compounds containing a C(O)NHP(O) skeleton, see: Toghraee *et al.* (2011 ▶). For their strengths and for structure determinations of CClF_2_C(O)NHP(O) compounds, see: Pourayoubi *et al.* (2011 ▶), and references cited therein. For bond lengths, angles and torsion angles in related structures, see: Tarahhomi *et al.* (2011 ▶). For hydrogen-bond motifs, see: Bernstein *et al.* (1995 ▶). For the synthesis of the starting material, CClF_2_C(O)NHP(O)Cl_2_, see: Iriarte *et al.* (2008 ▶).
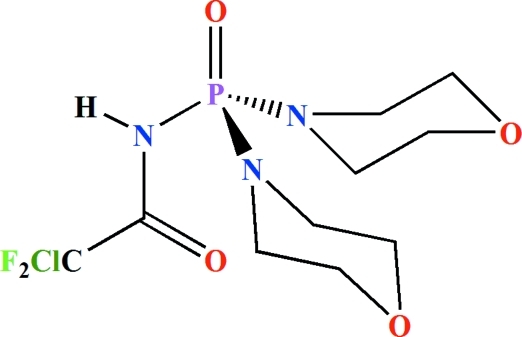


## Experimental

### 

#### Crystal data

C_10_H_17_ClF_2_N_3_O_4_P
*M* *_r_* = 347.69
Triclinic, 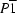

*a* = 7.6460 (11) Å
*b* = 12.5507 (18) Å
*c* = 16.477 (2) Å
α = 70.605 (3)°
β = 89.562 (3)°
γ = 82.155 (3)°
*V* = 1476.3 (4) Å^3^
*Z* = 4
Mo *K*α radiation
μ = 0.41 mm^−1^
*T* = 100 K
0.30 × 0.23 × 0.21 mm

#### Data collection

Bruker APEXII CCD area-detector diffractometer
Absorption correction: multi-scan (*SADABS*; Bruker, 2005 ▶) *T*_min_ = 0.887, *T*_max_ = 0.919
15034 measured reflections
6398 independent reflections
4124 reflections with *I* > 2σ(*I*)
*R*_int_ = 0.046

#### Refinement

*R*[*F* ^2^ > 2σ(*F* ^2^)] = 0.065
*wR*(*F* ^2^) = 0.172
*S* = 0.99
6398 reflections
490 parameters
H-atom parameters constrained
Δρ_max_ = 1.03 e Å^−3^
Δρ_min_ = −0.40 e Å^−3^

### 

Data collection: *APEX2* (Bruker, 2005 ▶); cell refinement: *SAINT* (Bruker, 2005 ▶); data reduction: *SAINT*; program(s) used to solve structure: *SHELXTL* (Sheldrick, 2008 ▶); program(s) used to refine structure: *SHELXTL*; molecular graphics: *SHELXTL*; software used to prepare material for publication: *SHELXTL*.

## Supplementary Material

Crystal structure: contains datablock(s) I, global. DOI: 10.1107/S1600536811030194/sj5185sup1.cif

Structure factors: contains datablock(s) I. DOI: 10.1107/S1600536811030194/sj5185Isup2.hkl

Supplementary material file. DOI: 10.1107/S1600536811030194/sj5185Isup3.cml

Additional supplementary materials:  crystallographic information; 3D view; checkCIF report

## Figures and Tables

**Table 1:** Hydrogen-bond geometry (Å, °)

*D*—H⋯*A*	*D*—H	H⋯*A*	*D*⋯*A*	*D*—H⋯*A*
N1—H1N⋯O2^i^	0.90	1.87	2.746 (4)	166
N1*A*—H1N*A*⋯O2*A*^ii^	0.90	1.89	2.731 (4)	154

Symmetry codes: (i) 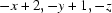; (ii) 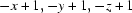.

**Fig. 1.**
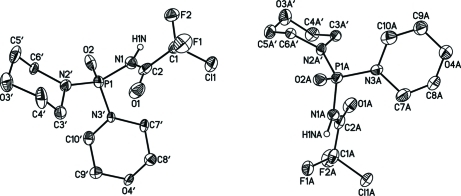
The asymmetric unit of the title compound with thermal ellipsoids drawn at the 50% probability level. Only the major disorder components are shown. With the exception of H1N and H1NA, hydrogen atoms are omitted for clarity.

**Fig. 2.**
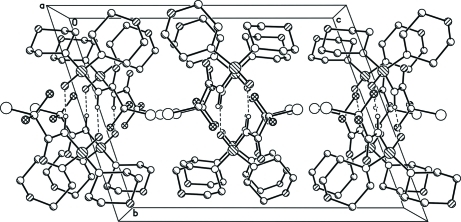
Crystal packing of the title compound, N—H···O hydrogen bonds are shown as dashed lines.

## supplementary crystallographic information

### Comment

In recent papers on phosphoric triamides containing a C(O)NHP(O) skeleton, patterns of hydrogen bonds (Toghraee *et al.*, 2011) and their strengths (Pourayoubi *et al.*, 2011) have been discussed.  The synthesis and X-ray crystal structure of the title phosphoric triamide is a continuation of work on this family of compounds in our laboratory. We have previously reported three derivatives with the CClF_2_C(O)NHP(O) unit (Pourayoubi *et al.*, 2011, and references cited therein).

Single crystals of the title compound, CClF_2_C(O)NHP(O)[NC_4_H_8_O]_2_, were obtained from CHCl_3_/n-C_7_H_16_ at room temperature. The asymmetric unit (Fig. 1) contains two independent molecules. Each of the two NC_4_H_8_O rings in the P1 phosphoramide is disordered over two sets of sites with site occupancies of 0.766 (7) and 0.234 (7) for N2'O3'/N2O3 ring and 0.639 (10) and 0.361 (10) for N3'O4'/N3O4 ring. Moreover, one of the NC_4_H_8_O moieties in P1A phosphoramide are disordered over two sets of sites with site occupancies of 0.807 (6) and 0.193 (6).

The P═O, C═O and P—N bond lengths, P—N—C bond angles and O—P—N—C torsion angles are within the expected values (Tarahhomi *et al.* 2011). The P═O and C═O groups are in *anti* positions with respect to each other. The P atom of each independent molecule is in a distorted tetrahedral environment as has been noted for other phosphoric triamides.

In each molecule, the phosphoryl group and the N—H unit are in a *syn* orientation with respect to one another and in the crystal, pairs of intermolecular N—H···O(P) hydrogen bonds (Table 1) form two independent centrosymmetric dimers (Fig. 2) as *R*_2_^2^(8) rings [for graph-set notation, see Bernstein *et al.* (1995)].

### Experimental

CClF_2_C(O)NHP(O)Cl_2_ was prepared according to procedure reported by Iriarte *et al.* (2008).

To a solution of CClF_2_C(O)NHP(O)Cl_2_ (2 mmol) in dry CHCl_3_, a solution of morpholine (8 mmol) in dry CHCl_3_ was added dropwise. After stirring for 4 h at 273 K, the solvent was evaporated at room temperature. The solid was washed with H_2_O. A crystalline product, suitable for X-ray crystallography, was obtained from a mixture of CHCl_3_/n-C_7_H_16_ (4:1) after slow evaporation at room temperature.

### Refinement

All hydrogen atoms were calculated from geometrical point of view with exception of H1N and H1NA, which were located from difference Fourier map. The H atoms were refined in isotropic approximation using a riding model with the *U*_iso_(H) parameters equal to 1.2 *U*_eq_(C,*N*), where U(C,*N*) are respectively the equivalent thermal parameters of the carbon and nitrogen atoms to which corresponding H atoms are bonded.

### Crystal data

**Table:**

C_10_H_17_ClF_2_N_3_O_4_P	*Z* = 4
*M**_r_* = 347.69	*F*(000) = 720
Triclinic, *P*1	*D*_x_ = 1.564 Mg m^−^^3^
Hall symbol: -P 1	Mo *K*α radiation, λ = 0.71073 Å
*a* = 7.6460 (11) Å	Cell parameters from 2709 reflections
*b* = 12.5507 (18) Å	θ = 2.6–25.0°
*c* = 16.477 (2) Å	µ = 0.41 mm^−^^1^
α = 70.605 (3)°	*T* = 100 K
β = 89.562 (3)°	Prism, colorless
γ = 82.155 (3)°	0.30 × 0.23 × 0.21 mm
*V* = 1476.3 (4) Å^3^	

### Data collection

**Table:**

Bruker APEXII CCD area-detector diffractometer	6398 independent reflections
Radiation source: fine-focus sealed tube	4124 reflections with *I* > 2σ(*I*)
graphite	*R*_int_ = 0.046
φ and ω scans	θ_max_ = 27.0°, θ_min_ = 1.3°
Absorption correction: multi-scan (*SADABS*; Bruker, 2005)	*h* = −9→9
*T*_min_ = 0.887, *T*_max_ = 0.919	*k* = −16→16
15034 measured reflections	*l* = −21→21

### Refinement

**Table:**

Refinement on *F*^2^	Primary atom site location: structure-invariant direct methods
Least-squares matrix: full	Secondary atom site location: difference Fourier map
*R*[*F*^2^ > 2σ(*F*^2^)] = 0.065	Hydrogen site location: inferred from neighbouring sites
*wR*(*F*^2^) = 0.172	H-atom parameters constrained
*S* = 0.99	*w* = 1/[σ^2^(*F*_o_^2^) + (0.0993*P*)^2^] where *P* = (*F*_o_^2^ + 2*F*_c_^2^)/3
6398 reflections	(Δ/σ)_max_ < 0.001
490 parameters	Δρ_max_ = 1.03 e Å^−^^3^
0 restraints	Δρ_min_ = −0.40 e Å^−^^3^

### Special details

**Table:**

Geometry. All e.s.d.'s (except the e.s.d. in the dihedral angle between two l.s. planes) are estimated using the full covariance matrix. The cell e.s.d.'s are taken into account individually in the estimation of e.s.d.'s in distances, angles and torsion angles; correlations between e.s.d.'s in cell parameters are only used when they are defined by crystal symmetry. An approximate (isotropic) treatment of cell e.s.d.'s is used for estimating e.s.d.'s involving l.s. planes.
Refinement. Refinement of *F*^2^ against ALL reflections. The weighted *R*-factor *wR* and goodness of fit *S* are based on *F*^2^, conventional *R*-factors *R* are based on *F*, with *F* set to zero for negative *F*^2^. The threshold expression of *F*^2^ > σ(*F*^2^) is used only for calculating *R*-factors(gt) *etc*. and is not relevant to the choice of reflections for refinement. *R*-factors based on *F*^2^ are statistically about twice as large as those based on *F*, and *R*- factors based on ALL data will be even larger.

### Fractional atomic coordinates and isotropic or equivalent isotropic displacement parameters (Å^2^)

**Table:**

	*x*	*y*	*z*	*U*_iso_*/*U*_eq_	Occ. (<1)
Cl1	0.64061 (15)	0.51615 (9)	0.20802 (6)	0.0404 (3)	
P1	0.97314 (14)	0.31136 (8)	0.04189 (6)	0.0270 (2)	
F1	0.3883 (3)	0.4651 (2)	0.13511 (16)	0.0449 (6)	
F2	0.5485 (3)	0.57780 (18)	0.04940 (13)	0.0323 (5)	
O1	0.6257 (4)	0.2874 (2)	0.14390 (17)	0.0395 (7)	
O2	1.0847 (3)	0.3817 (2)	−0.02205 (18)	0.0332 (6)	
N1	0.8158 (4)	0.4049 (2)	0.06533 (19)	0.0249 (7)	
H1N	0.8315	0.4788	0.0454	0.030*	
C1	0.5553 (5)	0.4848 (3)	0.1202 (2)	0.0298 (9)	
C2	0.6707 (5)	0.3800 (3)	0.1106 (2)	0.0266 (8)	
O3'	0.715 (3)	0.0970 (17)	−0.0749 (16)	0.039 (2)	0.766 (7)
N2'	0.8984 (8)	0.2232 (7)	0.0034 (6)	0.0246 (15)	0.766 (7)
C3'	0.8327 (8)	0.1183 (4)	0.0565 (3)	0.0311 (13)	0.766 (7)
H3'A	0.8026	0.1234	0.1137	0.037*	0.766 (7)
H3'B	0.9251	0.0518	0.0648	0.037*	0.766 (7)
C4'	0.6695 (10)	0.1036 (6)	0.0114 (5)	0.0399 (18)	0.766 (7)
H4'A	0.6241	0.0329	0.0464	0.048*	0.766 (7)
H4'B	0.5758	0.1689	0.0050	0.048*	0.766 (7)
C5'	0.7650 (11)	0.2044 (8)	−0.1253 (6)	0.037 (2)	0.766 (7)
H5'A	0.6674	0.2663	−0.1282	0.044*	0.766 (7)
H5'B	0.7878	0.2050	−0.1846	0.044*	0.766 (7)
C6'	0.9302 (7)	0.2243 (4)	−0.0849 (3)	0.0227 (12)	0.766 (7)
H6'A	1.0288	0.1639	−0.0841	0.027*	0.766 (7)
H6'B	0.9643	0.2988	−0.1198	0.027*	0.766 (7)
O3	0.687 (10)	0.114 (6)	−0.083 (6)	0.039 (2)	0.234 (7)
N2	0.846 (4)	0.241 (3)	−0.001 (2)	0.0246 (15)	0.234 (7)
C3	0.717 (3)	0.1595 (15)	0.0368 (11)	0.0311 (13)	0.234 (7)
H3A	0.7391	0.1272	0.1000	0.037*	0.234 (7)
H3B	0.5959	0.2016	0.0254	0.037*	0.234 (7)
C4	0.731 (4)	0.067 (2)	0.0019 (16)	0.040 (4)	0.234 (7)
H4A	0.8527	0.0261	0.0108	0.048*	0.234 (7)
H4B	0.6493	0.0128	0.0309	0.048*	0.234 (7)
C5	0.836 (4)	0.181 (2)	−0.126 (2)	0.038 (7)	0.234 (7)
H5A	0.9528	0.1334	−0.1076	0.046*	0.234 (7)
H5B	0.8219	0.2049	−0.1893	0.046*	0.234 (7)
C6	0.821 (3)	0.2784 (15)	−0.0976 (11)	0.034 (4)	0.234 (7)
H6A	0.7028	0.3240	−0.1155	0.040*	0.234 (7)
H6B	0.9110	0.3270	−0.1250	0.040*	0.234 (7)
O4'	1.2491 (14)	0.0934 (9)	0.2886 (5)	0.0339 (16)	0.639 (10)
N3'	1.0762 (11)	0.2376 (7)	0.1321 (5)	0.0185 (15)	0.639 (10)
C7'	1.0972 (9)	0.2777 (6)	0.2052 (4)	0.0247 (16)	0.639 (10)
H7'A	0.9951	0.3354	0.2055	0.030*	0.639 (10)
H7'B	1.2058	0.3140	0.1995	0.030*	0.639 (10)
C8'	1.1086 (10)	0.1794 (6)	0.2872 (4)	0.0268 (16)	0.639 (10)
H8'A	1.1255	0.2066	0.3362	0.032*	0.639 (10)
H8'B	0.9962	0.1470	0.2945	0.032*	0.639 (10)
C9'	1.2313 (11)	0.0521 (7)	0.2174 (5)	0.0350 (18)	0.639 (10)
H9'A	1.1214	0.0173	0.2222	0.042*	0.639 (10)
H9'B	1.3323	−0.0074	0.2195	0.042*	0.639 (10)
C10'	1.2261 (9)	0.1505 (6)	0.1317 (4)	0.0270 (16)	0.639 (10)
H10G	1.3380	0.1834	0.1251	0.032*	0.639 (10)
H10E	1.2107	0.1224	0.0830	0.032*	0.639 (10)
O4	1.293 (3)	0.1013 (19)	0.2677 (10)	0.0339 (16)	0.361 (10)
N3	1.019 (2)	0.2110 (13)	0.1428 (11)	0.027 (3)	0.361 (10)
C7	1.0145 (17)	0.2283 (11)	0.2273 (7)	0.029 (3)	0.361 (10)
H7A	0.9450	0.1734	0.2669	0.035*	0.361 (10)
H7B	0.9556	0.3063	0.2202	0.035*	0.361 (10)
C8	1.201 (2)	0.2118 (11)	0.2661 (7)	0.031 (3)	0.361 (10)
H8A	1.2658	0.2735	0.2311	0.037*	0.361 (10)
H8B	1.1955	0.2150	0.3253	0.037*	0.361 (10)
C9	1.309 (2)	0.0981 (12)	0.1852 (10)	0.041 (4)	0.361 (10)
H9A	1.3821	0.0260	0.1867	0.050*	0.361 (10)
H9B	1.3695	0.1622	0.1503	0.050*	0.361 (10)
C10	1.139 (2)	0.1062 (11)	0.1458 (8)	0.041 (4)	0.361 (10)
H10C	1.0830	0.0387	0.1784	0.050*	0.361 (10)
H10D	1.1541	0.1050	0.0864	0.050*	0.361 (10)
Cl1A	0.85569 (15)	0.48941 (9)	0.71485 (6)	0.0406 (3)	
P1A	0.51464 (13)	0.69026 (8)	0.44889 (6)	0.0247 (2)	
F1A	0.9510 (3)	0.43046 (19)	0.58641 (14)	0.0340 (5)	
F2A	1.1040 (3)	0.5467 (2)	0.61364 (16)	0.0484 (7)	
O1A	0.8595 (4)	0.7212 (2)	0.53451 (17)	0.0372 (7)	
O2A	0.4069 (3)	0.6174 (2)	0.42301 (16)	0.0282 (6)	
N1A	0.6740 (4)	0.5999 (2)	0.51829 (18)	0.0242 (7)	
H1NA	0.6824	0.5238	0.5440	0.029*	
C1A	0.9396 (5)	0.5230 (3)	0.6101 (2)	0.0295 (8)	
C2A	0.8199 (5)	0.6264 (3)	0.5487 (2)	0.0278 (8)	
O3A'	0.8020 (9)	0.8854 (9)	0.2277 (7)	0.0401 (17)	0.807 (6)
N2A'	0.5859 (6)	0.7791 (5)	0.3641 (4)	0.0215 (11)	0.807 (6)
C3A'	0.6568 (7)	0.8826 (4)	0.3614 (3)	0.0229 (10)	0.807 (6)
H3AC	0.6799	0.8813	0.4208	0.027*	0.807 (6)
H3AD	0.5695	0.9505	0.3323	0.027*	0.807 (6)
C4A'	0.8264 (8)	0.8900 (5)	0.3135 (3)	0.0331 (13)	0.807 (6)
H4AC	0.8701	0.9623	0.3093	0.040*	0.807 (6)
H4AD	0.9170	0.8262	0.3462	0.040*	0.807 (6)
C5A'	0.7448 (8)	0.7800 (5)	0.2351 (3)	0.0351 (13)	0.807 (6)
H5AC	0.8364	0.7169	0.2679	0.042*	0.807 (6)
H5AD	0.7313	0.7749	0.1768	0.042*	0.807 (6)
C6A'	0.5719 (8)	0.7657 (4)	0.2795 (3)	0.0244 (11)	0.807 (6)
H6AC	0.4766	0.8233	0.2438	0.029*	0.807 (6)
H6AD	0.5413	0.6890	0.2864	0.029*	0.807 (6)
O3A	0.756 (5)	0.902 (4)	0.217 (3)	0.0401 (17)	0.193 (6)
N2A	0.645 (3)	0.763 (3)	0.367 (2)	0.0215 (11)	0.193 (6)
C3A	0.758 (3)	0.8515 (17)	0.3631 (13)	0.0229 (10)	0.193 (6)
H3AA	0.7308	0.8819	0.4108	0.027*	0.193 (6)
H3AB	0.8839	0.8166	0.3706	0.027*	0.193 (6)
C4A	0.732 (4)	0.942 (2)	0.2832 (16)	0.040 (4)	0.193 (6)
H4AA	0.6105	0.9838	0.2789	0.048*	0.193 (6)
H4AB	0.8159	0.9969	0.2803	0.048*	0.193 (6)
C5A	0.661 (4)	0.822 (2)	0.2113 (15)	0.0351 (13)	0.193 (6)
H5AA	0.7028	0.7951	0.1632	0.042*	0.193 (6)
H5AB	0.5350	0.8545	0.1990	0.042*	0.193 (6)
C6A	0.679 (3)	0.726 (2)	0.2906 (13)	0.031 (5)	0.193 (6)
H6AA	0.8004	0.6839	0.2966	0.037*	0.193 (6)
H6AB	0.5954	0.6734	0.2885	0.037*	0.193 (6)
O4A	0.2244 (4)	0.9125 (2)	0.58234 (17)	0.0350 (6)	
N3A	0.4219 (4)	0.7723 (3)	0.50114 (19)	0.0303 (7)	
C7A	0.3976 (6)	0.7325 (3)	0.5954 (2)	0.0386 (10)	
H7AA	0.2944	0.6907	0.6085	0.046*	
H7AB	0.5034	0.6797	0.6254	0.046*	
C8A	0.3700 (6)	0.8304 (4)	0.6258 (2)	0.0390 (10)	
H8AA	0.3495	0.8031	0.6883	0.047*	
H8AB	0.4784	0.8672	0.6173	0.047*	
C9A	0.2413 (6)	0.9509 (4)	0.4929 (3)	0.0401 (10)	
H9AA	0.3445	0.9926	0.4788	0.048*	
H9AB	0.1347	1.0049	0.4652	0.048*	
C10A	0.2637 (6)	0.8563 (4)	0.4576 (3)	0.0389 (10)	
H10A	0.2800	0.8865	0.3948	0.047*	
H10B	0.1568	0.8180	0.4673	0.047*	

### Atomic displacement parameters (Å^2^)

**Table:**

	*U*^11^	*U*^22^	*U*^33^	*U*^12^	*U*^13^	*U*^23^
Cl1	0.0494 (6)	0.0469 (6)	0.0286 (5)	0.0007 (5)	−0.0053 (4)	−0.0201 (5)
P1	0.0368 (6)	0.0219 (5)	0.0251 (5)	−0.0013 (4)	−0.0059 (4)	−0.0127 (4)
F1	0.0254 (12)	0.0656 (17)	0.0439 (14)	−0.0116 (12)	0.0083 (11)	−0.0165 (13)
F2	0.0274 (11)	0.0381 (12)	0.0266 (11)	0.0003 (10)	−0.0006 (9)	−0.0066 (10)
O1	0.0531 (19)	0.0408 (17)	0.0268 (14)	−0.0212 (14)	0.0034 (13)	−0.0084 (13)
O2	0.0292 (14)	0.0298 (14)	0.0494 (17)	−0.0043 (11)	0.0028 (12)	−0.0248 (13)
N1	0.0253 (16)	0.0234 (15)	0.0273 (16)	−0.0049 (13)	0.0003 (13)	−0.0095 (13)
C1	0.025 (2)	0.042 (2)	0.0214 (18)	−0.0113 (17)	0.0012 (15)	−0.0064 (17)
C2	0.031 (2)	0.032 (2)	0.0149 (16)	−0.0071 (16)	−0.0038 (15)	−0.0047 (15)
O3'	0.040 (7)	0.042 (7)	0.048 (6)	−0.014 (4)	−0.002 (5)	−0.029 (5)
N2'	0.034 (5)	0.021 (3)	0.0191 (19)	−0.008 (3)	0.002 (3)	−0.006 (2)
C3'	0.051 (4)	0.022 (3)	0.022 (2)	−0.011 (2)	0.011 (2)	−0.007 (2)
C4'	0.046 (4)	0.044 (4)	0.049 (4)	−0.026 (3)	0.020 (3)	−0.034 (3)
C5'	0.035 (5)	0.054 (6)	0.030 (3)	−0.004 (4)	−0.005 (4)	−0.027 (4)
C6'	0.035 (3)	0.022 (3)	0.013 (2)	−0.008 (2)	0.004 (2)	−0.0084 (19)
O3	0.040 (7)	0.042 (7)	0.048 (6)	−0.014 (4)	−0.002 (5)	−0.029 (5)
N2	0.034 (5)	0.021 (3)	0.0191 (19)	−0.008 (3)	0.002 (3)	−0.006 (2)
C3	0.051 (4)	0.022 (3)	0.022 (2)	−0.011 (2)	0.011 (2)	−0.007 (2)
C4	0.049 (11)	0.043 (11)	0.039 (9)	−0.020 (9)	0.005 (8)	−0.022 (8)
C5	0.043 (17)	0.020 (11)	0.035 (11)	0.004 (11)	0.001 (13)	0.011 (9)
C6	0.047 (12)	0.029 (9)	0.027 (9)	0.005 (9)	0.010 (8)	−0.017 (8)
O4'	0.050 (6)	0.033 (2)	0.014 (4)	0.012 (3)	0.002 (3)	−0.009 (3)
N3'	0.025 (4)	0.017 (4)	0.017 (3)	−0.002 (3)	−0.002 (3)	−0.010 (3)
C7'	0.031 (4)	0.027 (3)	0.022 (3)	−0.005 (3)	−0.002 (3)	−0.016 (3)
C8'	0.027 (4)	0.027 (3)	0.026 (3)	−0.004 (3)	0.002 (3)	−0.009 (3)
C9'	0.044 (4)	0.031 (4)	0.026 (4)	0.007 (3)	−0.004 (3)	−0.009 (3)
C10'	0.027 (4)	0.030 (4)	0.029 (3)	−0.009 (3)	0.004 (3)	−0.015 (3)
O4	0.050 (6)	0.033 (2)	0.014 (4)	0.012 (3)	0.002 (3)	−0.009 (3)
N3	0.039 (10)	0.019 (7)	0.025 (6)	−0.006 (5)	0.001 (6)	−0.009 (5)
C7	0.039 (7)	0.029 (6)	0.021 (5)	−0.008 (6)	0.005 (5)	−0.010 (5)
C8	0.041 (8)	0.035 (7)	0.023 (6)	−0.004 (6)	0.001 (5)	−0.017 (5)
C9	0.065 (10)	0.025 (7)	0.034 (8)	0.010 (7)	0.001 (7)	−0.016 (6)
C10	0.065 (10)	0.024 (6)	0.036 (7)	−0.001 (7)	0.014 (7)	−0.012 (5)
Cl1A	0.0519 (6)	0.0464 (6)	0.0207 (5)	−0.0006 (5)	0.0053 (4)	−0.0099 (4)
P1A	0.0385 (6)	0.0177 (4)	0.0185 (4)	−0.0072 (4)	0.0021 (4)	−0.0055 (4)
F1A	0.0323 (12)	0.0418 (13)	0.0320 (12)	−0.0009 (10)	−0.0004 (10)	−0.0194 (11)
F2A	0.0290 (13)	0.0733 (18)	0.0486 (15)	−0.0176 (13)	−0.0010 (11)	−0.0239 (14)
O1A	0.0517 (18)	0.0369 (16)	0.0308 (15)	−0.0258 (14)	0.0055 (13)	−0.0142 (13)
O2A	0.0353 (15)	0.0225 (13)	0.0263 (13)	−0.0053 (11)	−0.0022 (11)	−0.0070 (11)
N1A	0.0311 (17)	0.0195 (15)	0.0232 (15)	−0.0081 (13)	0.0020 (13)	−0.0069 (12)
C1A	0.026 (2)	0.042 (2)	0.0267 (19)	−0.0108 (17)	0.0042 (15)	−0.0183 (18)
C2A	0.036 (2)	0.034 (2)	0.0195 (17)	−0.0134 (17)	0.0067 (15)	−0.0132 (16)
O3A'	0.048 (5)	0.040 (4)	0.037 (3)	−0.018 (3)	0.022 (3)	−0.014 (3)
N2A'	0.027 (3)	0.022 (2)	0.0202 (16)	−0.008 (3)	0.004 (3)	−0.0104 (17)
C3A'	0.026 (3)	0.021 (2)	0.022 (2)	−0.005 (2)	0.002 (2)	−0.0072 (18)
C4A'	0.032 (3)	0.034 (3)	0.034 (3)	−0.016 (2)	0.009 (2)	−0.008 (2)
C5A'	0.045 (4)	0.035 (3)	0.026 (3)	−0.006 (3)	0.012 (2)	−0.010 (2)
C6A'	0.032 (3)	0.020 (2)	0.020 (2)	0.001 (2)	0.001 (2)	−0.0064 (18)
O3A	0.048 (5)	0.040 (4)	0.037 (3)	−0.018 (3)	0.022 (3)	−0.014 (3)
N2A	0.027 (3)	0.022 (2)	0.0202 (16)	−0.008 (3)	0.004 (3)	−0.0104 (17)
C3A	0.026 (3)	0.021 (2)	0.022 (2)	−0.005 (2)	0.002 (2)	−0.0072 (18)
C4A	0.049 (11)	0.043 (11)	0.039 (9)	−0.020 (9)	0.005 (8)	−0.022 (8)
C5A	0.045 (4)	0.035 (3)	0.026 (3)	−0.006 (3)	0.012 (2)	−0.010 (2)
C6A	0.028 (12)	0.044 (13)	0.022 (10)	−0.002 (11)	0.012 (9)	−0.015 (10)
O4A	0.0404 (16)	0.0337 (15)	0.0293 (14)	−0.0037 (13)	0.0061 (12)	−0.0090 (12)
N3A	0.047 (2)	0.0214 (15)	0.0187 (15)	0.0015 (14)	−0.0018 (14)	−0.0041 (13)
C7A	0.054 (3)	0.034 (2)	0.024 (2)	−0.007 (2)	0.0035 (18)	−0.0046 (17)
C8A	0.047 (3)	0.044 (2)	0.0223 (19)	−0.001 (2)	0.0075 (18)	−0.0086 (18)
C9A	0.033 (2)	0.049 (3)	0.031 (2)	0.0049 (19)	0.0033 (18)	−0.009 (2)
C10A	0.041 (2)	0.038 (2)	0.033 (2)	−0.0036 (19)	−0.0034 (18)	−0.0075 (19)

### Geometric parameters (Å, °)

**Table:**

Cl1—C1	1.771 (4)	C8—H8A	0.9900
P1—O2	1.480 (3)	C8—H8B	0.9900
P1—N3'	1.610 (9)	C9—C10	1.44 (2)
P1—N2'	1.612 (9)	C9—H9A	0.9900
P1—N1	1.698 (3)	C9—H9B	0.9900
P1—N2	1.70 (4)	C10—H10C	0.9900
P1—N3	1.723 (16)	C10—H10D	0.9900
F1—C1	1.338 (4)	Cl1A—C1A	1.773 (4)
F2—C1	1.344 (4)	P1A—O2A	1.471 (3)
O1—C2	1.205 (4)	P1A—N2A'	1.616 (6)
N1—C2	1.346 (5)	P1A—N3A	1.639 (3)
N1—H1N	0.8999	P1A—N1A	1.692 (3)
C1—C2	1.534 (6)	P1A—N2A	1.75 (3)
O3'—C5'	1.43 (3)	F1A—C1A	1.334 (4)
O3'—C4'	1.49 (3)	F2A—C1A	1.337 (4)
N2'—C3'	1.469 (10)	O1A—C2A	1.215 (4)
N2'—C6'	1.469 (10)	N1A—C2A	1.351 (5)
C3'—C4'	1.520 (9)	N1A—H1NA	0.8998
C3'—H3'A	0.9900	C1A—C2A	1.538 (6)
C3'—H3'B	0.9900	O3A'—C5A'	1.416 (12)
C4'—H4'A	0.9900	O3A'—C4A'	1.448 (12)
C4'—H4'B	0.9900	N2A'—C3A'	1.462 (7)
C5'—C6'	1.520 (10)	N2A'—C6A'	1.466 (8)
C5'—H5'A	0.9900	C3A'—C4A'	1.511 (7)
C5'—H5'B	0.9900	C3A'—H3AC	0.9900
C6'—H6'A	0.9900	C3A'—H3AD	0.9900
C6'—H6'B	0.9900	C4A'—H4AC	0.9900
O3—C4	1.35 (9)	C4A'—H4AD	0.9900
O3—C5	1.54 (10)	C5A'—C6A'	1.510 (8)
N2—C3	1.50 (4)	C5A'—H5AC	0.9900
N2—C6	1.51 (4)	C5A'—H5AD	0.9900
C3—C4	1.44 (3)	C6A'—H6AC	0.9900
C3—H3A	0.9900	C6A'—H6AD	0.9900
C3—H3B	0.9900	O3A—C4A	1.35 (6)
C4—H4A	0.9900	O3A—C5A	1.35 (6)
C4—H4B	0.9900	N2A—C3A	1.49 (4)
C5—C6	1.44 (4)	N2A—C6A	1.49 (4)
C5—H5A	0.9900	C3A—C4A	1.42 (3)
C5—H5B	0.9900	C3A—H3AA	0.9900
C6—H6A	0.9900	C3A—H3AB	0.9900
C6—H6B	0.9900	C4A—H4AA	0.9900
O4'—C8'	1.409 (11)	C4A—H4AB	0.9900
O4'—C9'	1.446 (12)	C5A—C6A	1.44 (3)
N3'—C7'	1.469 (10)	C5A—H5AA	0.9900
N3'—C10'	1.474 (11)	C5A—H5AB	0.9900
C7'—C8'	1.491 (10)	C6A—H6AA	0.9900
C7'—H7'A	0.9900	C6A—H6AB	0.9900
C7'—H7'B	0.9900	O4A—C9A	1.401 (5)
C8'—H8'A	0.9900	O4A—C8A	1.423 (5)
C8'—H8'B	0.9900	N3A—C7A	1.484 (5)
C9'—C10'	1.534 (11)	N3A—C10A	1.500 (5)
C9'—H9'A	0.9900	C7A—C8A	1.463 (6)
C9'—H9'B	0.9900	C7A—H7AA	0.9900
C10'—H10G	0.9900	C7A—H7AB	0.9900
C10'—H10E	0.9900	C8A—H8AA	0.9900
O4—C9	1.38 (2)	C8A—H8AB	0.9900
O4—C8	1.46 (2)	C9A—C10A	1.476 (6)
N3—C7	1.48 (2)	C9A—H9AA	0.9900
N3—C10	1.48 (2)	C9A—H9AB	0.9900
C7—C8	1.53 (2)	C10A—H10A	0.9900
C7—H7A	0.9900	C10A—H10B	0.9900
C7—H7B	0.9900		
			
O2—P1—N3'	113.8 (3)	C7—C8—H8A	109.9
O2—P1—N2'	109.4 (3)	O4—C8—H8B	109.9
N3'—P1—N2'	107.4 (4)	C7—C8—H8B	109.9
O2—P1—N1	105.94 (15)	H8A—C8—H8B	108.3
N3'—P1—N1	105.6 (3)	O4—C9—C10	110.9 (15)
N2'—P1—N1	114.9 (2)	O4—C9—H9A	109.5
O2—P1—N2	113.0 (12)	C10—C9—H9A	109.5
N3'—P1—N2	116.1 (11)	O4—C9—H9B	109.5
N1—P1—N2	100.7 (9)	C10—C9—H9B	109.5
O2—P1—N3	132.9 (5)	H9A—C9—H9B	108.0
N2'—P1—N3	93.5 (6)	C9—C10—N3	113.1 (12)
N1—P1—N3	100.2 (6)	C9—C10—H10C	109.0
N2—P1—N3	99.4 (13)	N3—C10—H10C	109.0
C2—N1—P1	126.8 (3)	C9—C10—H10D	109.0
C2—N1—H1N	116.8	N3—C10—H10D	109.0
P1—N1—H1N	116.3	H10C—C10—H10D	107.8
F1—C1—F2	106.4 (3)	O2A—P1A—N2A'	109.5 (2)
F1—C1—C2	110.9 (3)	O2A—P1A—N3A	118.81 (17)
F2—C1—C2	113.0 (3)	N2A'—P1A—N3A	103.5 (2)
F1—C1—Cl1	108.9 (3)	O2A—P1A—N1A	105.77 (14)
F2—C1—Cl1	108.6 (3)	N2A'—P1A—N1A	115.0 (2)
C2—C1—Cl1	109.0 (3)	N3A—P1A—N1A	104.59 (15)
O1—C2—N1	127.8 (4)	O2A—P1A—N2A	113.4 (11)
O1—C2—C1	118.6 (3)	N3A—P1A—N2A	111.7 (10)
N1—C2—C1	113.6 (3)	N1A—P1A—N2A	100.1 (9)
C5'—O3'—C4'	107.6 (15)	C2A—N1A—P1A	127.4 (3)
C3'—N2'—C6'	112.1 (6)	C2A—N1A—H1NA	103.5
C3'—N2'—P1	123.9 (6)	P1A—N1A—H1NA	129.1
C6'—N2'—P1	122.4 (6)	F1A—C1A—F2A	106.9 (3)
N2'—C3'—C4'	108.9 (5)	F1A—C1A—C2A	113.2 (3)
N2'—C3'—H3'A	109.9	F2A—C1A—C2A	110.6 (3)
C4'—C3'—H3'A	109.9	F1A—C1A—Cl1A	108.5 (3)
N2'—C3'—H3'B	109.9	F2A—C1A—Cl1A	108.4 (3)
C4'—C3'—H3'B	109.9	C2A—C1A—Cl1A	109.0 (3)
H3'A—C3'—H3'B	108.3	O1A—C2A—N1A	126.8 (4)
O3'—C4'—C3'	109.6 (9)	O1A—C2A—C1A	119.1 (3)
O3'—C4'—H4'A	109.8	N1A—C2A—C1A	114.1 (3)
C3'—C4'—H4'A	109.8	C5A'—O3A'—C4A'	108.4 (7)
O3'—C4'—H4'B	109.8	C3A'—N2A'—C6A'	113.4 (5)
C3'—C4'—H4'B	109.8	C3A'—N2A'—P1A	125.1 (4)
H4'A—C4'—H4'B	108.2	C6A'—N2A'—P1A	121.3 (4)
O3'—C5'—C6'	109.7 (10)	N2A'—C3A'—C4A'	109.8 (4)
O3'—C5'—H5'A	109.7	N2A'—C3A'—H3AC	109.7
C6'—C5'—H5'A	109.7	C4A'—C3A'—H3AC	109.7
O3'—C5'—H5'B	109.7	N2A'—C3A'—H3AD	109.7
C6'—C5'—H5'B	109.7	C4A'—C3A'—H3AD	109.7
H5'A—C5'—H5'B	108.2	H3AC—C3A'—H3AD	108.2
N2'—C6'—C5'	110.3 (5)	O3A'—C4A'—C3A'	111.9 (5)
N2'—C6'—H6'A	109.6	O3A'—C4A'—H4AC	109.2
C5'—C6'—H6'A	109.6	C3A'—C4A'—H4AC	109.2
N2'—C6'—H6'B	109.6	O3A'—C4A'—H4AD	109.2
C5'—C6'—H6'B	109.6	C3A'—C4A'—H4AD	109.2
H6'A—C6'—H6'B	108.1	H4AC—C4A'—H4AD	107.9
C4—O3—C5	107 (5)	O3A'—C5A'—C6A'	112.7 (5)
C3—N2—C6	107 (2)	O3A'—C5A'—H5AC	109.1
C3—N2—P1	133 (2)	C6A'—C5A'—H5AC	109.1
C6—N2—P1	119 (2)	O3A'—C5A'—H5AD	109.1
C4—C3—N2	112 (2)	C6A'—C5A'—H5AD	109.1
C4—C3—H3A	109.1	H5AC—C5A'—H5AD	107.8
N2—C3—H3A	109.1	N2A'—C6A'—C5A'	110.1 (4)
C4—C3—H3B	109.1	N2A'—C6A'—H6AC	109.7
N2—C3—H3B	109.1	C5A'—C6A'—H6AC	109.7
H3A—C3—H3B	107.9	N2A'—C6A'—H6AD	109.7
O3—C4—C3	107 (4)	C5A'—C6A'—H6AD	109.7
O3—C4—H4A	110.2	H6AC—C6A'—H6AD	108.2
C3—C4—H4A	110.2	C4A—O3A—C5A	119 (4)
O3—C4—H4B	110.2	C3A—N2A—C6A	108 (2)
C3—C4—H4B	110.2	C3A—N2A—P1A	131 (2)
H4A—C4—H4B	108.5	C6A—N2A—P1A	120 (2)
C6—C5—O3	106 (4)	C4A—C3A—N2A	111 (2)
C6—C5—H5A	110.6	C4A—C3A—H3AA	109.4
O3—C5—H5A	110.6	N2A—C3A—H3AA	109.4
C6—C5—H5B	110.6	C4A—C3A—H3AB	109.4
O3—C5—H5B	110.6	N2A—C3A—H3AB	109.4
H5A—C5—H5B	108.8	H3AA—C3A—H3AB	108.0
C5—C6—N2	110 (2)	O3A—C4A—C3A	111 (3)
C5—C6—H6A	109.6	O3A—C4A—H4AA	109.5
N2—C6—H6A	109.6	C3A—C4A—H4AA	109.5
C5—C6—H6B	109.6	O3A—C4A—H4AB	109.5
N2—C6—H6B	109.6	C3A—C4A—H4AB	109.5
H6A—C6—H6B	108.1	H4AA—C4A—H4AB	108.1
C8'—O4'—C9'	111.2 (8)	O3A—C5A—C6A	111 (3)
C7'—N3'—C10'	110.2 (6)	O3A—C5A—H5AA	109.5
C7'—N3'—P1	125.4 (6)	C6A—C5A—H5AA	109.5
C10'—N3'—P1	118.1 (6)	O3A—C5A—H5AB	109.5
N3'—C7'—C8'	109.6 (5)	C6A—C5A—H5AB	109.5
N3'—C7'—H7'A	109.8	H5AA—C5A—H5AB	108.1
C8'—C7'—H7'A	109.8	C5A—C6A—N2A	112 (2)
N3'—C7'—H7'B	109.8	C5A—C6A—H6AA	109.2
C8'—C7'—H7'B	109.8	N2A—C6A—H6AA	109.2
H7'A—C7'—H7'B	108.2	C5A—C6A—H6AB	109.2
O4'—C8'—C7'	111.8 (6)	N2A—C6A—H6AB	109.2
O4'—C8'—H8'A	109.3	H6AA—C6A—H6AB	107.9
C7'—C8'—H8'A	109.3	C9A—O4A—C8A	112.0 (3)
O4'—C8'—H8'B	109.3	C7A—N3A—C10A	108.6 (3)
C7'—C8'—H8'B	109.3	C7A—N3A—P1A	123.9 (3)
H8'A—C8'—H8'B	107.9	C10A—N3A—P1A	116.7 (2)
O4'—C9'—C10'	110.2 (7)	C8A—C7A—N3A	109.7 (3)
O4'—C9'—H9'A	109.6	C8A—C7A—H7AA	109.7
C10'—C9'—H9'A	109.6	N3A—C7A—H7AA	109.7
O4'—C9'—H9'B	109.6	C8A—C7A—H7AB	109.7
C10'—C9'—H9'B	109.6	N3A—C7A—H7AB	109.7
H9'A—C9'—H9'B	108.1	H7AA—C7A—H7AB	108.2
N3'—C10'—C9'	107.8 (6)	O4A—C8A—C7A	112.7 (4)
N3'—C10'—H10G	110.2	O4A—C8A—H8AA	109.1
C9'—C10'—H10G	110.2	C7A—C8A—H8AA	109.1
N3'—C10'—H10E	110.2	O4A—C8A—H8AB	109.1
C9'—C10'—H10E	110.2	C7A—C8A—H8AB	109.1
H10G—C10'—H10E	108.5	H8AA—C8A—H8AB	107.8
C9—O4—C8	110.2 (15)	O4A—C9A—C10A	112.2 (4)
C7—N3—C10	111.6 (13)	O4A—C9A—H9AA	109.2
C7—N3—P1	128.5 (12)	C10A—C9A—H9AA	109.2
C10—N3—P1	115.6 (11)	O4A—C9A—H9AB	109.2
N3—C7—C8	111.0 (10)	C10A—C9A—H9AB	109.2
N3—C7—H7A	109.4	H9AA—C9A—H9AB	107.9
C8—C7—H7A	109.4	C9A—C10A—N3A	109.5 (3)
N3—C7—H7B	109.4	C9A—C10A—H10A	109.8
C8—C7—H7B	109.4	N3A—C10A—H10A	109.8
H7A—C7—H7B	108.0	C9A—C10A—H10B	109.8
O4—C8—C7	109.0 (13)	N3A—C10A—H10B	109.8
O4—C8—H8A	109.9	H10A—C10A—H10B	108.2
			
O2—P1—N1—C2	169.6 (3)	N3'—P1—N3—C10	−79 (2)
N3'—P1—N1—C2	−69.4 (4)	N2'—P1—N3—C10	56.8 (11)
N2'—P1—N1—C2	48.7 (5)	N1—P1—N3—C10	172.8 (10)
N2—P1—N1—C2	51.7 (13)	N2—P1—N3—C10	70.0 (14)
N3—P1—N1—C2	−50.0 (6)	C10—N3—C7—C8	46.6 (15)
P1—N1—C2—O1	1.1 (5)	P1—N3—C7—C8	−108.7 (15)
P1—N1—C2—C1	179.9 (2)	C9—O4—C8—C7	62.3 (19)
F1—C1—C2—O1	−24.5 (5)	N3—C7—C8—O4	−53.1 (15)
F2—C1—C2—O1	−143.8 (3)	C8—O4—C9—C10	−65 (2)
Cl1—C1—C2—O1	95.3 (3)	O4—C9—C10—N3	58 (2)
F1—C1—C2—N1	156.5 (3)	C7—N3—C10—C9	−48.7 (17)
F2—C1—C2—N1	37.2 (4)	P1—N3—C10—C9	110.0 (15)
Cl1—C1—C2—N1	−83.6 (3)	O2A—P1A—N1A—C2A	168.7 (3)
O2—P1—N2'—C3'	159.7 (4)	N2A'—P1A—N1A—C2A	47.8 (4)
N3'—P1—N2'—C3'	35.7 (6)	N3A—P1A—N1A—C2A	−65.1 (3)
N1—P1—N2'—C3'	−81.3 (5)	N2A—P1A—N1A—C2A	50.6 (11)
N2—P1—N2'—C3'	−93 (6)	P1A—N1A—C2A—O1A	3.5 (5)
N3—P1—N2'—C3'	21.6 (7)	P1A—N1A—C2A—C1A	−179.1 (2)
O2—P1—N2'—C6'	−4.5 (5)	F1A—C1A—C2A—O1A	−144.0 (3)
N3'—P1—N2'—C6'	−128.5 (5)	F2A—C1A—C2A—O1A	−24.0 (5)
N1—P1—N2'—C6'	114.4 (4)	Cl1A—C1A—C2A—O1A	95.1 (4)
N2—P1—N2'—C6'	103 (6)	F1A—C1A—C2A—N1A	38.4 (4)
N3—P1—N2'—C6'	−142.6 (7)	F2A—C1A—C2A—N1A	158.4 (3)
C6'—N2'—C3'—C4'	−54.5 (6)	Cl1A—C1A—C2A—N1A	−82.5 (3)
P1—N2'—C3'—C4'	139.8 (5)	O2A—P1A—N2A'—C3A'	163.3 (4)
C5'—O3'—C4'—C3'	−64.5 (11)	N3A—P1A—N2A'—C3A'	35.6 (4)
N2'—C3'—C4'—O3'	59.1 (10)	N1A—P1A—N2A'—C3A'	−77.9 (4)
C4'—O3'—C5'—C6'	63.6 (12)	N2A—P1A—N2A'—C3A'	−89 (5)
C3'—N2'—C6'—C5'	54.4 (7)	O2A—P1A—N2A'—C6A'	−12.1 (4)
P1—N2'—C6'—C5'	−139.7 (6)	N3A—P1A—N2A'—C6A'	−139.8 (4)
O3'—C5'—C6'—N2'	−59.2 (12)	N1A—P1A—N2A'—C6A'	106.7 (4)
O2—P1—N2—C3	179 (2)	N2A—P1A—N2A'—C6A'	96 (5)
N3'—P1—N2—C3	45 (3)	C6A'—N2A'—C3A'—C4A'	−51.0 (6)
N2'—P1—N2—C3	100 (7)	P1A—N2A'—C3A'—C4A'	133.3 (4)
N1—P1—N2—C3	−69 (2)	C5A'—O3A'—C4A'—C3A'	−60.7 (6)
N3—P1—N2—C3	34 (3)	N2A'—C3A'—C4A'—O3A'	55.9 (7)
O2—P1—N2—C6	−16 (2)	C4A'—O3A'—C5A'—C6A'	60.5 (6)
N3'—P1—N2—C6	−150.0 (15)	C3A'—N2A'—C6A'—C5A'	50.5 (5)
N2'—P1—N2—C6	−94 (6)	P1A—N2A'—C6A'—C5A'	−133.6 (4)
N1—P1—N2—C6	96.7 (18)	O3A'—C5A'—C6A'—N2A'	−55.7 (7)
N3—P1—N2—C6	−160.9 (18)	O2A—P1A—N2A—C3A	174 (2)
C6—N2—C3—C4	53 (3)	N2A'—P1A—N2A—C3A	96 (5)
P1—N2—C3—C4	−140 (2)	N3A—P1A—N2A—C3A	36 (3)
C5—O3—C4—C3	70 (5)	N1A—P1A—N2A—C3A	−74 (2)
N2—C3—C4—O3	−63 (5)	O2A—P1A—N2A—C6A	−17 (2)
C4—O3—C5—C6	−71 (5)	N2A'—P1A—N2A—C6A	−95 (5)
O3—C5—C6—N2	61 (4)	N3A—P1A—N2A—C6A	−154.5 (17)
C3—N2—C6—C5	−54 (3)	N1A—P1A—N2A—C6A	95 (2)
P1—N2—C6—C5	137 (2)	C6A—N2A—C3A—C4A	55 (3)
O2—P1—N3'—C7'	87.1 (7)	P1A—N2A—C3A—C4A	−135 (2)
N2'—P1—N3'—C7'	−151.7 (6)	C5A—O3A—C4A—C3A	55 (4)
N1—P1—N3'—C7'	−28.7 (7)	N2A—C3A—C4A—O3A	−55 (3)
N2—P1—N3'—C7'	−139.2 (12)	C4A—O3A—C5A—C6A	−53 (4)
N3—P1—N3'—C7'	−105 (3)	O3A—C5A—C6A—N2A	50 (3)
O2—P1—N3'—C10'	−62.4 (6)	C3A—N2A—C6A—C5A	−53 (3)
N2'—P1—N3'—C10'	58.9 (6)	P1A—N2A—C6A—C5A	136 (2)
N1—P1—N3'—C10'	−178.1 (5)	O2A—P1A—N3A—C7A	82.6 (4)
N2—P1—N3'—C10'	71.3 (12)	N2A'—P1A—N3A—C7A	−155.8 (4)
N3—P1—N3'—C10'	105 (3)	N1A—P1A—N3A—C7A	−35.0 (4)
C10'—N3'—C7'—C8'	−58.6 (8)	N2A—P1A—N3A—C7A	−142.4 (10)
P1—N3'—C7'—C8'	150.0 (7)	O2A—P1A—N3A—C10A	−57.6 (3)
C9'—O4'—C8'—C7'	−58.1 (9)	N2A'—P1A—N3A—C10A	64.0 (3)
N3'—C7'—C8'—O4'	57.7 (9)	N1A—P1A—N3A—C10A	−175.2 (3)
C8'—O4'—C9'—C10'	58.5 (9)	N2A—P1A—N3A—C10A	77.4 (10)
C7'—N3'—C10'—C9'	58.8 (7)	C10A—N3A—C7A—C8A	−56.7 (5)
P1—N3'—C10'—C9'	−147.4 (6)	P1A—N3A—C7A—C8A	160.3 (3)
O4'—C9'—C10'—N3'	−58.3 (8)	C9A—O4A—C8A—C7A	−56.0 (5)
O2—P1—N3—C7	90.5 (14)	N3A—C7A—C8A—O4A	56.7 (5)
N3'—P1—N3—C7	75 (2)	C8A—O4A—C9A—C10A	56.1 (5)
N2'—P1—N3—C7	−148.8 (13)	O4A—C9A—C10A—N3A	−57.2 (5)
N1—P1—N3—C7	−32.7 (13)	C7A—N3A—C10A—C9A	56.9 (4)
N2—P1—N3—C7	−135.5 (15)	P1A—N3A—C10A—C9A	−157.2 (3)
O2—P1—N3—C10	−63.9 (13)		

### Hydrogen-bond geometry (Å, °)

**Table:**

*D*—H···*A*	*D*—H	H···*A*	*D*···*A*	*D*—H···*A*
N1—H1N···O2^i^	0.90	1.87	2.746 (4)	166
N1A—H1NA···O2A^ii^	0.90	1.89	2.731 (4)	154

Symmetry codes: (i) −*x*+2, −*y*+1, −*z*; (ii) −*x*+1, −*y*+1, −*z*+1.
